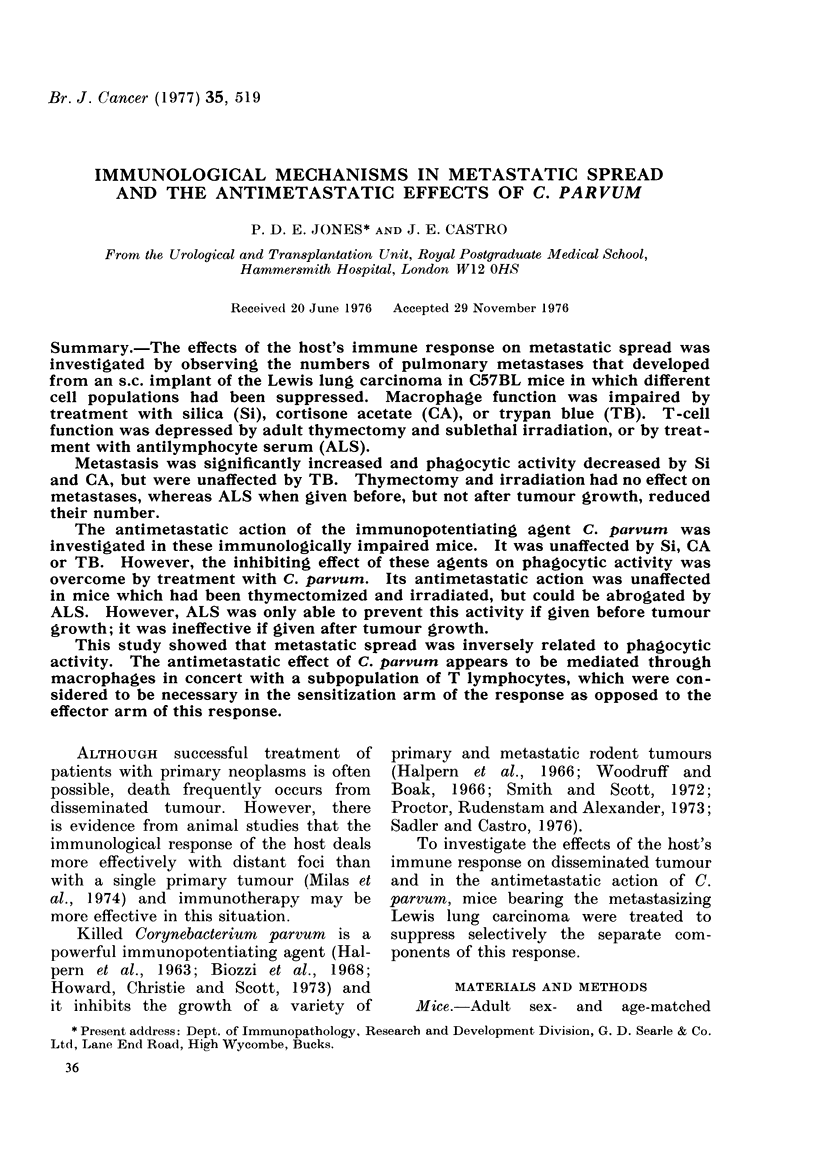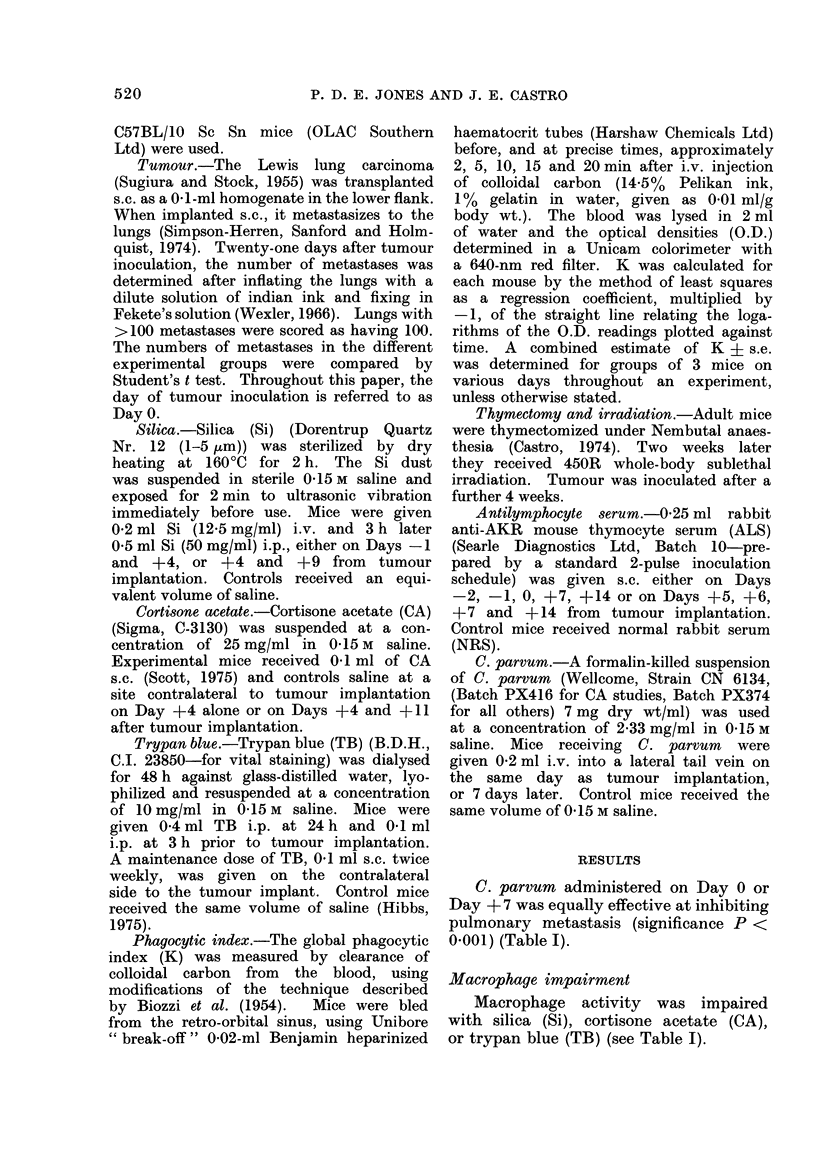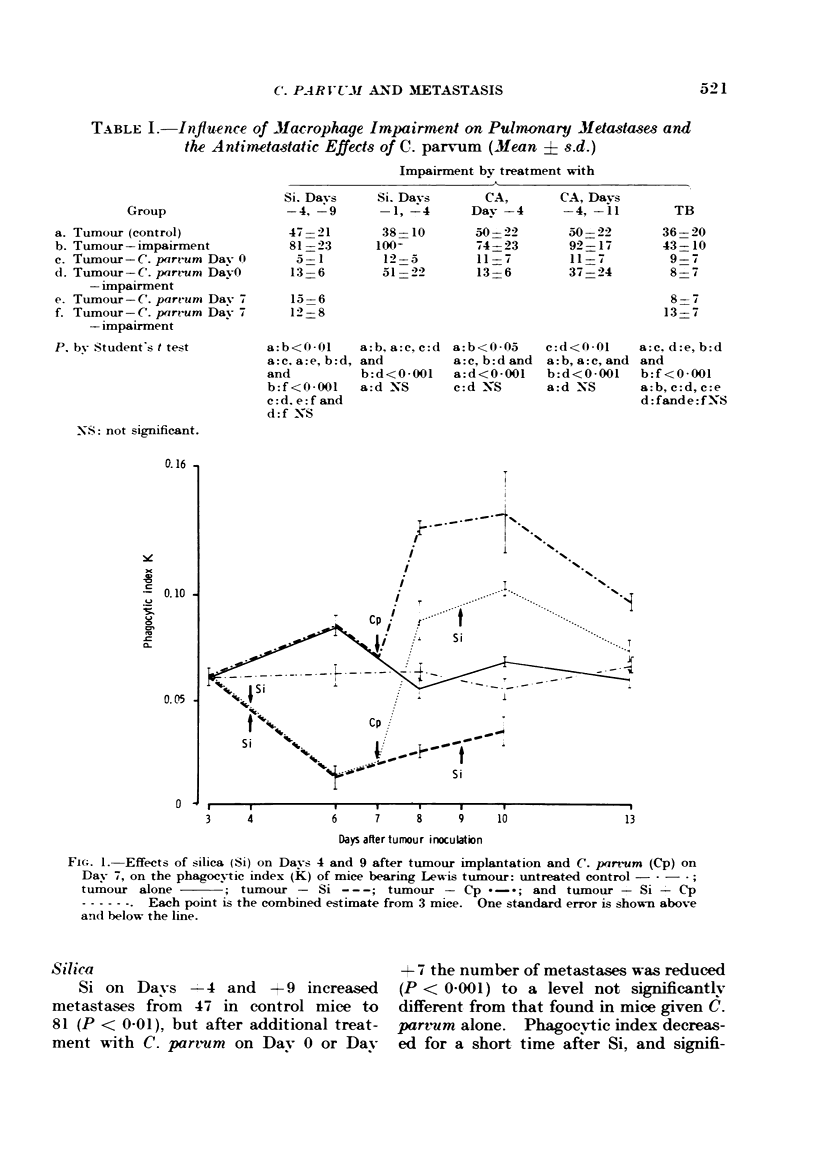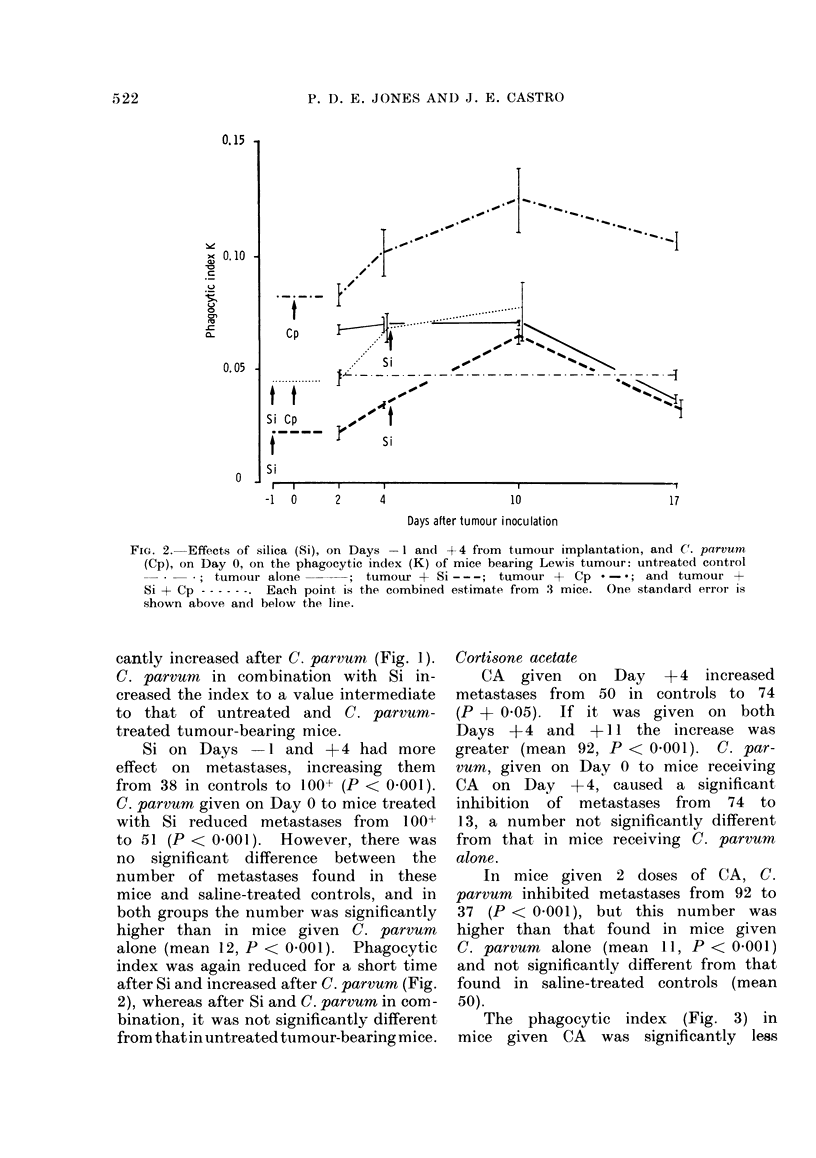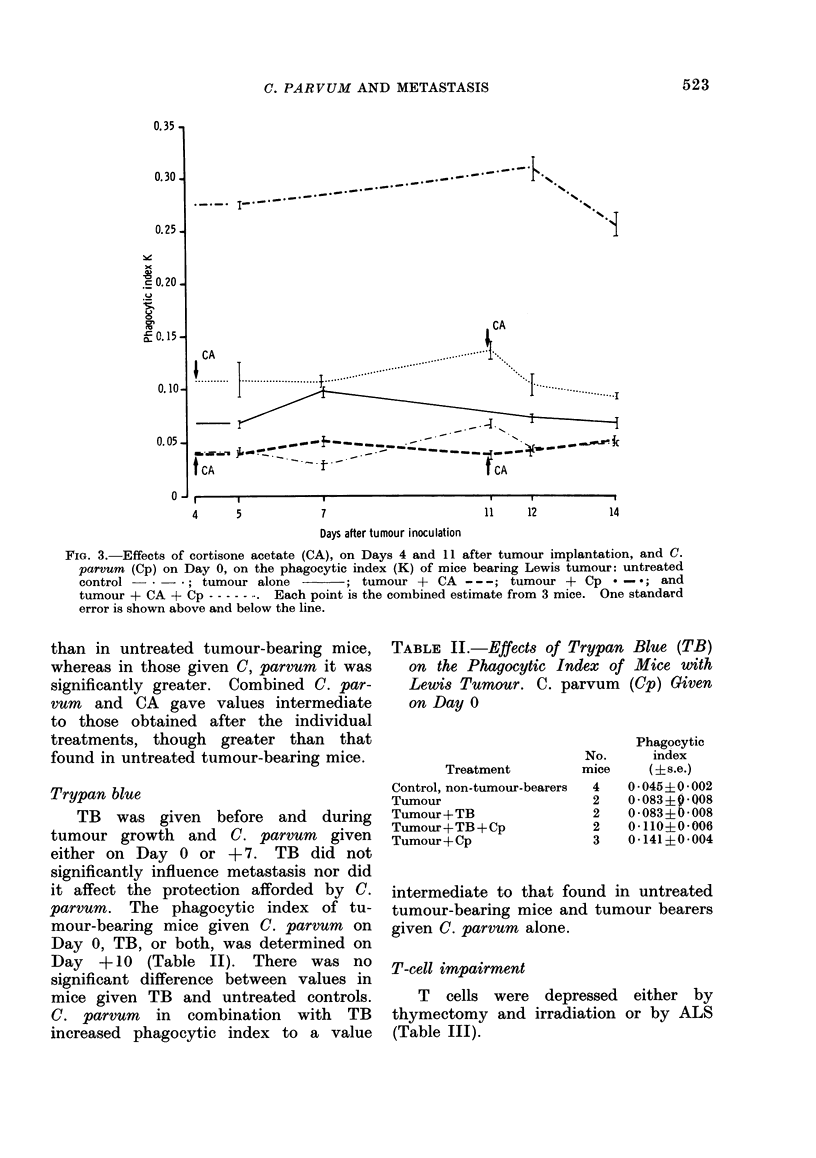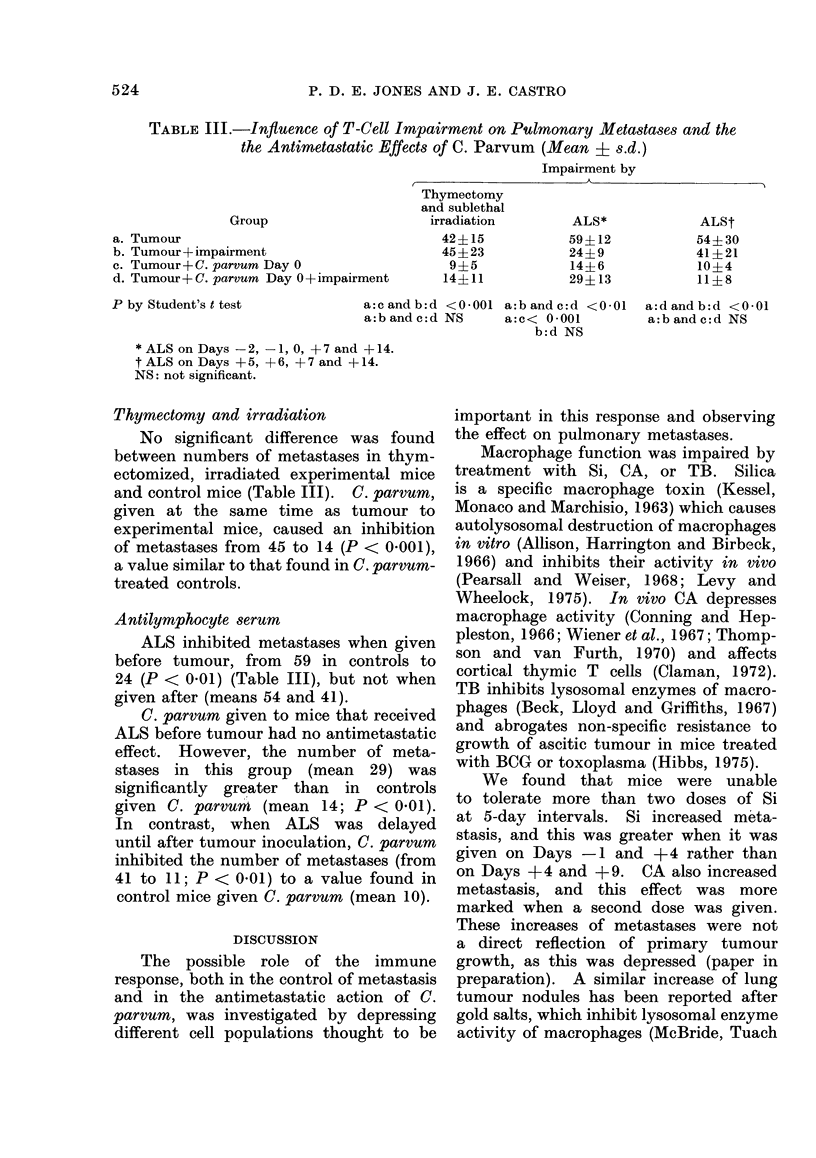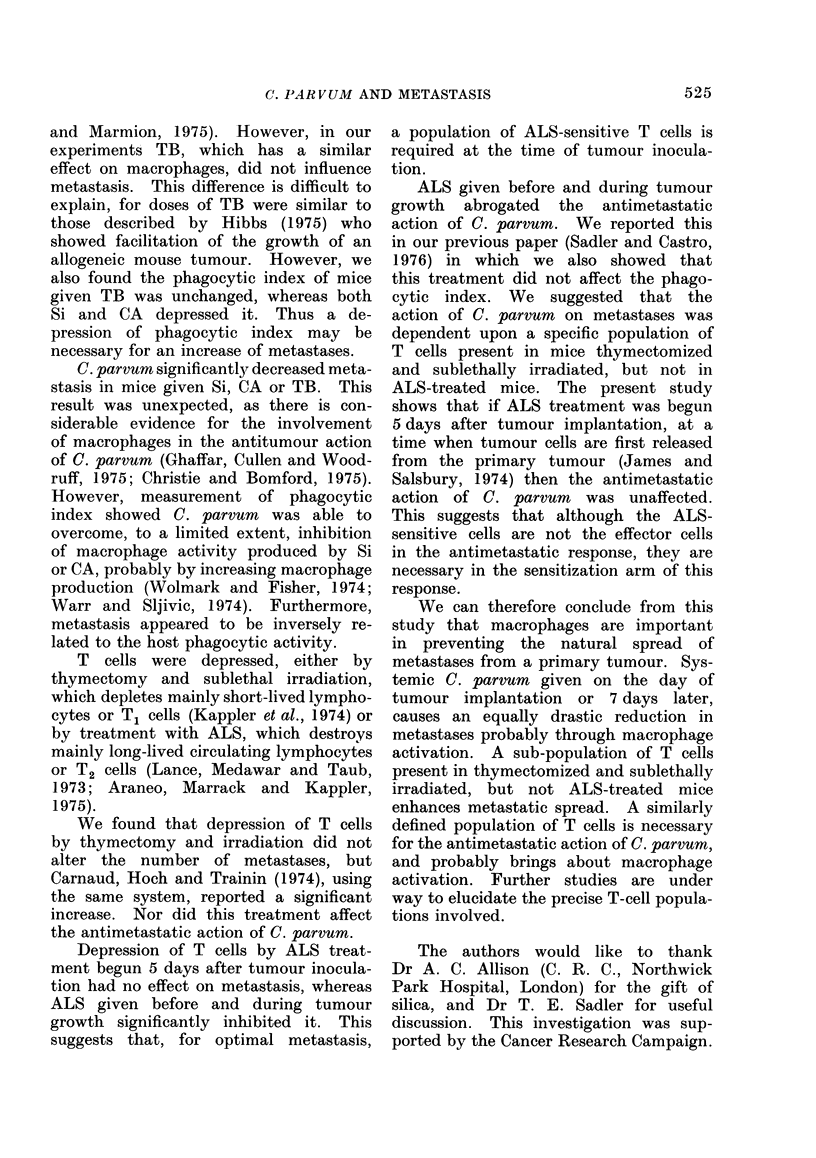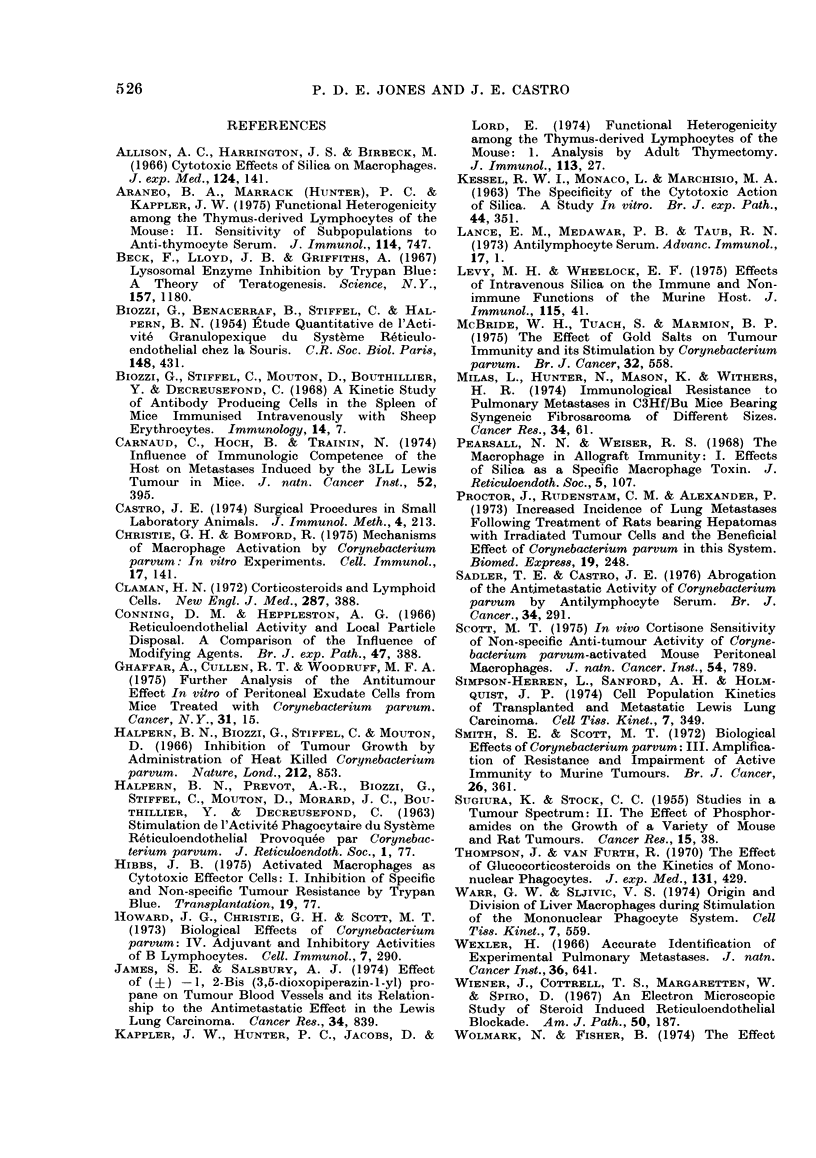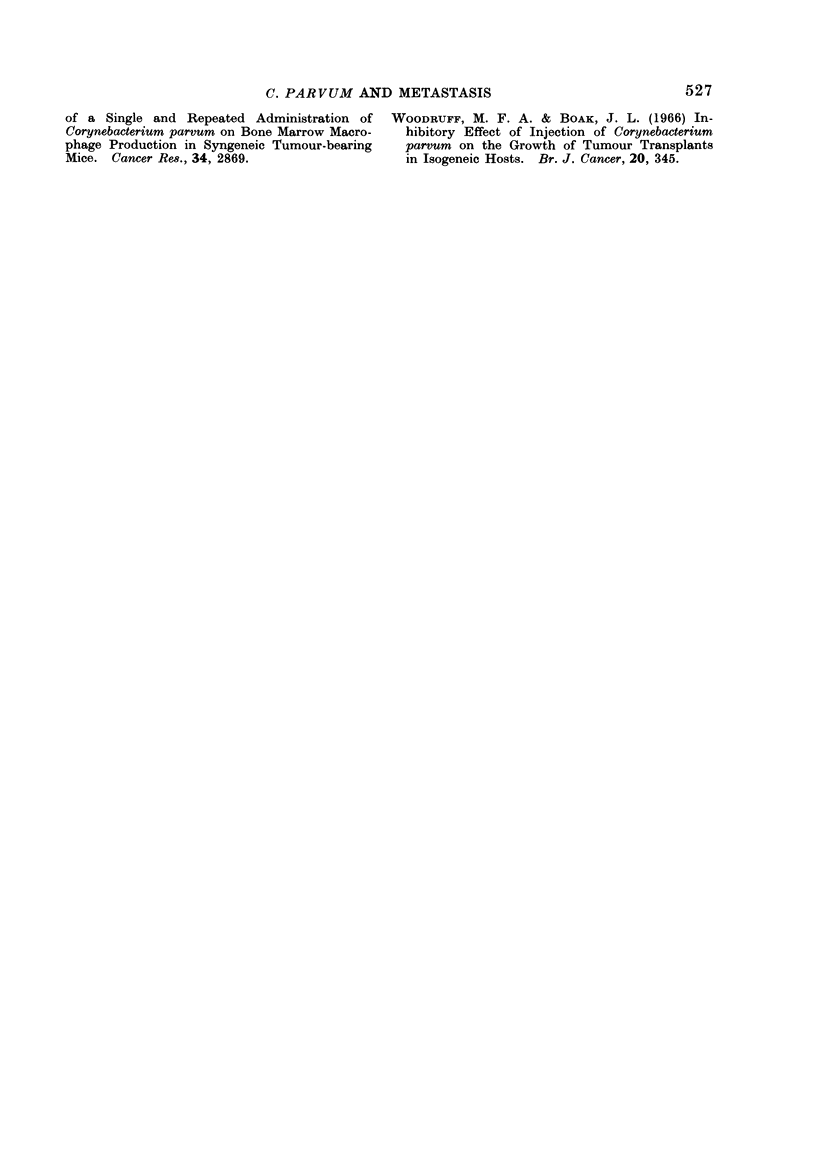# Immunological mechanisms in metastatic spread and the antimetastatic effects of C. parvum.

**DOI:** 10.1038/bjc.1977.85

**Published:** 1977-05

**Authors:** P. D. Jones, J. E. Castro

## Abstract

The effects of the host's immune response on metastatic spread was investigated by observing the numbers of pulmonary metastases that developed from an s.c. implant of the Lewis lung carcinoma in C57BL mice in which different cell populations had been suppressed. Macrophage function was impaired by treatment with silica (Si), cortisone acetate (CA), or trypan blue (TB). T-cell function was depressed by adult thymectomy and sublethal irradiation, or by treatment with antilymphocyte serum (ALS). Metastasis was significantly increased and phagocytic activity decreased by Si and CA, but were unaffected by TB. Thymectomy and irradiation had no effect on metastases, whereas ALS when given before, but not after tumour growth, reduced their number. The antimetastatic action of the immunopotentiating agent C. parvum was investigated in these immunologically impaired mice. It was unaffected by Si, CA or TB. However, the inhibiting effect of these agents on phagocytic activity was overcome by treatment with C. parvum. Its antimetastatic action was unaffected in mice which had been thymectomized and irradiated, but could be abrogated by ALS. However, ALS was only able to prevent this activity if given before tumour growth; it was ineffective if given after tumour growth. This study showed that metastatic spread was inversely related to phagocytic activity. The antimetastatic effect of C. parvum appears to be mediated through macrophages in concert with a subpopulation of T lymphocytes, which were considered to be necessary in the sensitization arm of the response as opposed to the effector arm of this response.


*Br. J. Cancer* (1977) **35**, 519

### IMMUNOLOGICAL MECHANISMS IN METASTATIC SPREAD AND THE ANTIMETASTATIC EFFECTS OF *C. PARVUM*

P. D. E. JONES* AND J. E. CASTRO

*From the Urological and Transplantation Unit, Royal Postgraduate Medical School, Hammersmith Hospital, London W12 0HS*

Received 20 June 1976 Accepted 29 November 1976

**Summary.—The effects of the host's immune response on metastatic spread was investigated by observing the numbers of pulmonary metastases that developed from an s.c. implant of the Lewis lung carcinoma in C57BL mice in which different cell populations had been suppressed. Macrophage function was impaired by treatment with silica (Si), cortisone acetate (CA), or trypan blue (TB). T-cell function was depressed by adult thymectomy and sublethal irradiation, or by treatment with antilymphocyte serum (ALS).**

**Metastasis was significantly increased and phagocytic activity decreased by Si and CA, but were unaffected by TB. Thymectomy and irradiation had no effect on metastases, whereas ALS when given before, but not after tumour growth, reduced their number.**

**The antimetastatic action of the immunopotentiating agent *C. parvum* was investigated in these immunologically impaired mice. It was unaffected by Si, CA or TB. However, the inhibiting effect of these agents on phagocytic activity was overcome by treatment with *C. parvum*. Its antimetastatic action was unaffected in mice which had been thymectomized and irradiated, but could be abrogated by ALS. However, ALS was only able to prevent this activity if given before tumour growth; it was ineffective if given after tumour growth.**

**This study showed that metastatic spread was inversely related to phagocytic activity. The antimetastatic effect of *C. parvum* appears to be mediated through macrophages in concert with a subpopulation of T lymphocytes, which were considered to be necessary in the sensitization arm of the response as opposed to the effector arm of this response.**

ALTHOUGH successful treatment of patients with primary neoplasms is often possible, death frequently occurs from disseminated tumour. However, there is evidence from animal studies that the immunological response of the host deals more effectively with distant foci than with a single primary tumour (Milas *et al.*, 1974) and immunotherapy may be more effective in this situation.

Killed *Corynebacterium parvum* is a powerful immunopotentiating agent (Halpern *et al.*, 1963; Biozzi *et al.*, 1968; Howard, Christie and Scott, 1973) and it inhibits the growth of a variety of primary and metastatic rodent tumours (Halpern *et al.*, 1966; Woodruff and Boak, 1966; Smith and Scott, 1972; Proctor, Rudenstam and Alexander, 1973; Sadler and Castro, 1976).

To investigate the effects of the host's immune response on disseminated tumour and in the antimetastatic action of *C. parvum*, mice bearing the metastasizing Lewis lung carcinoma were treated to suppress selectively the separate components of this response.

### MATERIALS AND METHODS

*Mice.*—Adult sex- and age-matched

* Present address: Dept. of Immunopathology, Research and Development Division, G. D. Searle & Co. Ltd, Lane End Road, High Wycombe, Bucks.

C57BL/10 Sc Sn mice (OLAC Southern Ltd) were used.

*Tumour*.—The Lewis lung carcinoma (Sugiura and Stock, 1955) was transplanted s.c. as a 0·1-ml homogenate in the lower flank. When implanted s.c., it metastasizes to the lungs (Simpson-Herren, Sanford and Holmquist, 1974). Twenty-one days after tumour inoculation, the number of metastases was determined after inflating the lungs with a dilute solution of indian ink and fixing in Fekete's solution (Wexler, 1966). Lungs with >100 metastases were scored as having 100. The numbers of metastases in the different experimental groups were compared by Student's *t* test. Throughout this paper, the day of tumour inoculation is referred to as Day 0.

*Silica*.—Silica (Si) (Dorentrup Quartz Nr. 12 (1–5 μm)) was sterilized by dry heating at 160°C for 2 h. The Si dust was suspended in sterile 0·15 M saline and exposed for 2 min to ultrasonic vibration immediately before use. Mice were given 0·2 ml Si (12·5 mg/ml) i.v. and 3 h later 0·5 ml Si (50 mg/ml) i.p., either on Days −1 and +4, or +4 and +9 from tumour implantation. Controls received an equivalent volume of saline.

*Cortisone acetate*.—Cortisone acetate (CA) (Sigma, C-3130) was suspended at a concentration of 25 mg/ml in 0·15 M saline. Experimental mice received 0·1 ml of CA s.c. (Scott, 1975) and controls saline at a site contralateral to tumour implantation on Day +4 alone or on Days +4 and +11 after tumour implantation.

*Trypan blue*.—Trypan blue (TB) (B.D.H., C.I. 23850—for vital staining) was dialysed for 48 h against glass-distilled water, lyophilized and resuspended at a concentration of 10 mg/ml in 0·15 M saline. Mice were given 0·4 ml TB i.p. at 24 h and 0·1 ml i.p. at 3 h prior to tumour implantation. A maintenance dose of TB, 0·1 ml s.c. twice weekly, was given on the contralateral side to the tumour implant. Control mice received the same volume of saline (Hibbs, 1975).

*Phagocytic index*.—The global phagocytic index (K) was measured by clearance of colloidal carbon from the blood, using modifications of the technique described by Biozzi *et al.* (1954). Mice were bled from the retro-orbital sinus, using Unibore "break-off" 0·02-ml Benjamin heparinized haematocrit tubes (Harshaw Chemicals Ltd) before, and at precise times, approximately 2, 5, 10, 15 and 20 min after i.v. injection of colloidal carbon (14·5% Pelikan ink, 1% gelatin in water, given as 0·01 ml/g body wt.). The blood was lysed in 2 ml of water and the optical densities (O.D.) determined in a Unicam colorimeter with a 640-nm red filter. K was calculated for each mouse by the method of least squares as a regression coefficient, multiplied by −1, of the straight line relating the logarithms of the O.D. readings plotted against time. A combined estimate of K ± s.e. was determined for groups of 3 mice on various days throughout an experiment, unless otherwise stated.

*Thymectomy and irradiation*.—Adult mice were thymectomized under Nembutal anaesthesia (Castro, 1974). Two weeks later they received 450R whole-body sublethal irradiation. Tumour was inoculated after a further 4 weeks.

*Antilymphocyte serum*.—0·25 ml rabbit anti-AKR mouse thymocyte serum (ALS) (Searle Diagnostics Ltd, Batch 10—prepared by a standard 2-pulse inoculation schedule) was given s.c. either on Days −2, −1, 0, +7, +14 or on Days +5, +6, +7 and +14 from tumour implantation. Control mice received normal rabbit serum (NRS).

*C. parvum*.—A formalin-killed suspension of *C. parvum* (Wellcome, Strain CN 6134, (Batch PX416 for CA studies, Batch PX374 for all others) 7 mg dry wt/ml) was used at a concentration of 2·33 mg/ml in 0·15 M saline. Mice receiving *C. parvum* were given 0·2 ml i.v. into a lateral tail vein on the same day as tumour implantation, or 7 days later. Control mice received the same volume of 0·15 M saline.

### RESULTS

*C. parvum* administered on Day 0 or Day +7 was equally effective at inhibiting pulmonary metastasis (significance $P <$ 0·001) (Table I).

### *Macrophage impairment*

Macrophage activity was impaired with silica (Si), cortisone acetate (CA), or trypan blue (TB) (see Table I).

TABLE I.—*Influence of Macrophage Impairment on Pulmonary Metastases and the Antimetastatic Effects of* C. parvum (*Mean* ± *s.d.*)

| Group | Impairment by treatment with Si, Days –4, –9 | Si, Days –1, –4 | CA, Day –4 | CA, Days –4, –11 | TB |
|---|---|---|---|---|---|
| a. Tumour (control) | 47±21 | 38±10 | 50±22 | 50±22 | 36±20 |
| b. Tumour – impairment | 81±23 | 100– | 74±23 | 92±17 | 43±10 |
| c. Tumour – *C. parvum* Day 0 | 5±1 | 12±5 | 11±7 | 11±7 | 9±7 |
| d. Tumour – *C. parvum* Day0 – impairment | 13±6 | 51±22 | 13±6 | 37±24 | 8±7 |
| e. Tumour – *C. parvum* Day 7 | 15±6 | | | | 8±7 |
| f. Tumour – *C. parvum* Day 7 – impairment | 12±8 | | | | 13±7 |
| *P*, by Student's *t* test | a:b<0·01 a:c, a:e, b:d, and b:f<0·001 c:d, e:f and d:f NS | a:b, a:c, c:d and b:d<0·001 a:d NS | a:b<0·05 a:c, b:d and a:d<0·001 c:d NS | c:d<0·01 a:b, a:c, and b:d<0·001 a:d NS | a:c, d:e, b:d and b:f<0·001 a:b, c:d, c:e d:fande:fNS |

NS: not significant.

FIG. 1.—Effects of silica (Si) on Days 4 and 9 after tumour implantation and *C. parvum* (Cp) on Day 7, on the phagocytic index (K) of mice bearing Lewis tumour: untreated control — · — ·; tumour alone ————; tumour – Si – – –; tumour – Cp •—•; and tumour – Si – Cp - - - - - -. Each point is the combined estimate from 3 mice. One standard error is shown above and below the line.

*Silica*

Si on Days +4 and +9 increased metastases from 47 in control mice to 81 ($P < 0{\cdot}01$), but after additional treatment with *C. parvum* on Day 0 or Day +7 the number of metastases was reduced ($P < 0{\cdot}001$) to a level not significantly different from that found in mice given *C. parvum* alone. Phagocytic index decreased for a short time after Si, and signifi-

FIG. 2.—Effects of silica (Si), on Days −1 and +4 from tumour implantation, and *C. parvum* (Cp), on Day 0, on the phagocytic index (K) of mice bearing Lewis tumour: untreated control — · — ·; tumour alone ———; tumour + Si - - -; tumour + Cp • — •; and tumour + Si + Cp - - - - - -. Each point is the combined estimate from 3 mice. One standard error is shown above and below the line.

cantly increased after *C. parvum* (Fig. 1). *C. parvum* in combination with Si increased the index to a value intermediate to that of untreated and *C. parvum*-treated tumour-bearing mice.

Si on Days −1 and +4 had more effect on metastases, increasing them from 38 in controls to 100+ ($P < 0{\cdot}001$). *C. parvum* given on Day 0 to mice treated with Si reduced metastases from 100+ to 51 ($P < 0{\cdot}001$). However, there was no significant difference between the number of metastases found in these mice and saline-treated controls, and in both groups the number was significantly higher than in mice given *C. parvum* alone (mean 12, $P < 0{\cdot}001$). Phagocytic index was again reduced for a short time after Si and increased after *C. parvum* (Fig. 2), whereas after Si and *C. parvum* in combination, it was not significantly different from that in untreated tumour-bearing mice.

*Cortisone acetate*

CA given on Day +4 increased metastases from 50 in controls to 74 ($P + 0{\cdot}05$). If it was given on both Days +4 and +11 the increase was greater (mean 92, $P < 0{\cdot}001$). *C. parvum*, given on Day 0 to mice receiving CA on Day +4, caused a significant inhibition of metastases from 74 to 13, a number not significantly different from that in mice receiving *C. parvum alone*.

In mice given 2 doses of CA, *C. parvum* inhibited metastases from 92 to 37 ($P < 0{\cdot}001$), but this number was higher than that found in mice given *C. parvum* alone (mean 11, $P < 0{\cdot}001$) and not significantly different from that found in saline-treated controls (mean 50).

The phagocytic index (Fig. 3) in mice given CA was significantly less

FIG. 3.—Effects of cortisone acetate (CA), on Days 4 and 11 after tumour implantation, and *C. parvum* (Cp) on Day 0, on the phagocytic index (K) of mice bearing Lewis tumour: untreated control — · — ·; tumour alone ———; tumour + CA - - -; tumour + Cp • — •; and tumour + CA + Cp ·······. Each point is the combined estimate from 3 mice. One standard error is shown above and below the line.

than in untreated tumour-bearing mice, whereas in those given *C, parvum* it was significantly greater. Combined *C. parvum* and CA gave values intermediate to those obtained after the individual treatments, though greater than that found in untreated tumour-bearing mice.

### *Trypan blue*

TB was given before and during tumour growth and *C. parvum* given either on Day 0 or +7. TB did not significantly influence metastasis nor did it affect the protection afforded by *C. parvum*. The phagocytic index of tumour-bearing mice given *C. parvum* on Day 0, TB, or both, was determined on Day +10 (Table II). There was no significant difference between values in mice given TB and untreated controls. *C. parvum* in combination with TB increased phagocytic index to a value intermediate to that found in untreated tumour-bearing mice and tumour bearers given *C. parvum* alone.

TABLE II.—*Effects of Trypan Blue (TB) on the Phagocytic Index of Mice with Lewis Tumour.* C. parvum *(Cp) Given on Day* 0

| Treatment | No. mice | Phagocytic index (±s.e.) |
|---|---|---|
| Control, non-tumour-bearers | 4 | 0·045±0·002 |
| Tumour | 2 | 0·083±0·008 |
| Tumour+TB | 2 | 0·083±0·008 |
| Tumour+TB+Cp | 2 | 0·110±0·006 |
| Tumour+Cp | 3 | 0·141±0·004 |

### *T-cell impairment*

T cells were depressed either by thymectomy and irradiation or by ALS (Table III).

TABLE III.—*Influence of T-Cell Impairment on Pulmonary Metastases and the the Antimetastatic Effects of* C. Parvum (*Mean* ± *s.d.*)

| Group | Impairment by: Thymectomy and sublethal irradiation | ALS* | ALS† |
|---|---|---|---|
| a. Tumour | 42±15 | 59±12 | 54±30 |
| b. Tumour+impairment | 45±23 | 24±9 | 41±21 |
| c. Tumour+*C. parvum* Day 0 | 9±5 | 14±6 | 10±4 |
| d. Tumour+*C. parvum* Day 0+impairment | 14±11 | 29±13 | 11±8 |
| *P* by Student's *t* test | a:c and b:d <0·001; a:b and c:d NS | a:b and c:d <0·01; a:c< 0·001; b:d NS | a:d and b:d <0·01; a:b and c:d NS |

* ALS on Days −2, −1, 0, +7 and +14.

† ALS on Days +5, +6, +7 and +14.

NS: not significant.

*Thymectomy and irradiation*

No significant difference was found between numbers of metastases in thymectomized, irradiated experimental mice and control mice (Table III). *C. parvum*, given at the same time as tumour to experimental mice, caused an inhibition of metastases from 45 to 14 ($P < 0{\cdot}001$), a value similar to that found in *C. parvum*-treated controls.

*Antilymphocyte serum*

ALS inhibited metastases when given before tumour, from 59 in controls to 24 ($P < 0{\cdot}01$) (Table III), but not when given after (means 54 and 41).

*C. parvum* given to mice that received ALS before tumour had no antimetastatic effect. However, the number of metastases in this group (mean 29) was significantly greater than in controls given *C. parvum* (mean 14; $P < 0{\cdot}01$). In contrast, when ALS was delayed until after tumour inoculation, *C. parvum* inhibited the number of metastases (from 41 to 11; $P < 0{\cdot}01$) to a value found in control mice given *C. parvum* (mean 10).

### DISCUSSION

The possible role of the immune response, both in the control of metastasis and in the antimetastatic action of *C. parvum*, was investigated by depressing different cell populations thought to be important in this response and observing the effect on pulmonary metastases.

Macrophage function was impaired by treatment with Si, CA, or TB. Silica is a specific macrophage toxin (Kessel, Monaco and Marchisio, 1963) which causes autolysosomal destruction of macrophages *in vitro* (Allison, Harrington and Birbeck, 1966) and inhibits their activity *in vivo* (Pearsall and Weiser, 1968; Levy and Wheelock, 1975). *In vivo* CA depresses macrophage activity (Conning and Heppleston, 1966; Wiener *et al.*, 1967; Thompson and van Furth, 1970) and affects cortical thymic T cells (Claman, 1972). TB inhibits lysosomal enzymes of macrophages (Beck, Lloyd and Griffiths, 1967) and abrogates non-specific resistance to growth of ascitic tumour in mice treated with BCG or toxoplasma (Hibbs, 1975).

We found that mice were unable to tolerate more than two doses of Si at 5-day intervals. Si increased metastasis, and this was greater when it was given on Days −1 and +4 rather than on Days +4 and +9. CA also increased metastasis, and this effect was more marked when a second dose was given. These increases of metastases were not a direct reflection of primary tumour growth, as this was depressed (paper in preparation). A similar increase of lung tumour nodules has been reported after gold salts, which inhibit lysosomal enzyme activity of macrophages (McBride, Tuach and Marmion, 1975). However, in our experiments TB, which has a similar effect on macrophages, did not influence metastasis. This difference is difficult to explain, for doses of TB were similar to those described by Hibbs (1975) who showed facilitation of the growth of an allogeneic mouse tumour. However, we also found the phagocytic index of mice given TB was unchanged, whereas both Si and CA depressed it. Thus a depression of phagocytic index may be necessary for an increase of metastases.

*C. parvum* significantly decreased metastasis in mice given Si, CA or TB. This result was unexpected, as there is considerable evidence for the involvement of macrophages in the antitumour action of *C. parvum* (Ghaffar, Cullen and Woodruff, 1975; Christie and Bomford, 1975). However, measurement of phagocytic index showed *C. parvum* was able to overcome, to a limited extent, inhibition of macrophage activity produced by Si or CA, probably by increasing macrophage production (Wolmark and Fisher, 1974; Warr and Sljivic, 1974). Furthermore, metastasis appeared to be inversely related to the host phagocytic activity.

T cells were depressed, either by thymectomy and sublethal irradiation, which depletes mainly short-lived lymphocytes or $T_1$ cells (Kappler *et al.*, 1974) or by treatment with ALS, which destroys mainly long-lived circulating lymphocytes or $T_2$ cells (Lance, Medawar and Taub, 1973; Araneo, Marrack and Kappler, 1975).

We found that depression of T cells by thymectomy and irradiation did not alter the number of metastases, but Carnaud, Hoch and Trainin (1974), using the same system, reported a significant increase. Nor did this treatment affect the antimetastatic action of *C. parvum*.

Depression of T cells by ALS treatment begun 5 days after tumour inoculation had no effect on metastasis, whereas ALS given before and during tumour growth significantly inhibited it. This suggests that, for optimal metastasis, a population of ALS-sensitive T cells is required at the time of tumour inoculation.

ALS given before and during tumour growth abrogated the antimetastatic action of *C. parvum*. We reported this in our previous paper (Sadler and Castro, 1976) in which we also showed that this treatment did not affect the phagocytic index. We suggested that the action of *C. parvum* on metastases was dependent upon a specific population of T cells present in mice thymectomized and sublethally irradiated, but not in ALS-treated mice. The present study shows that if ALS treatment was begun 5 days after tumour implantation, at a time when tumour cells are first released from the primary tumour (James and Salsbury, 1974) then the antimetastatic action of *C. parvum* was unaffected. This suggests that although the ALS-sensitive cells are not the effector cells in the antimetastatic response, they are necessary in the sensitization arm of this response.

We can therefore conclude from this study that macrophages are important in preventing the natural spread of metastases from a primary tumour. Systemic *C. parvum* given on the day of tumour implantation or 7 days later, causes an equally drastic reduction in metastases probably through macrophage activation. A sub-population of T cells present in thymectomized and sublethally irradiated, but not ALS-treated mice enhances metastatic spread. A similarly defined population of T cells is necessary for the antimetastatic action of *C. parvum*, and probably brings about macrophage activation. Further studies are under way to elucidate the precise T-cell populations involved.

The authors would like to thank Dr A. C. Allison (C. R. C., Northwick Park Hospital, London) for the gift of silica, and Dr T. E. Sadler for useful discussion. This investigation was supported by the Cancer Research Campaign.

### REFERENCES

ALLISON, A. C., HARRINGTON, J. S. & BIRBECK, M. (1966) Cytotoxic Effects of Silica on Macrophages. *J. exp. Med.*, **124**, 141.

ARANEO, B. A., MARRACK (HUNTER), P. C. & KAPPLER, J. W. (1975) Functional Heterogenicity among the Thymus-derived Lymphocytes of the Mouse: II. Sensitivity of Subpopulations to Anti-thymocyte Serum. *J. Immunol.*, **114**, 747.

BECK, F., LLOYD, J. B. & GRIFFITHS, A. (1967) Lysosomal Enzyme Inhibition by Trypan Blue: A Theory of Teratogenesis. *Science, N.Y.*, **157**, 1180.

BIOZZI, G., BENACERRAF, B., STIFFEL, C. & HALPERN, B. N. (1954) Étude Quantitative de l'Activité Granulopexique du Système Réticulo-endothelial chez la Souris. *C.R. Soc. Biol. Paris*, **148**, 431.

BIOZZI, G., STIFFEL, C., MOUTON, D., BOUTHILLIER, Y. & DECREUSEFOND, C. (1968) A Kinetic Study of Antibody Producing Cells in the Spleen of Mice Immunised Intravenously with Sheep Erythrocytes. *Immunology*, **14**, 7.

CARNAUD, C., HOCH, B. & TRAININ, N. (1974) Influence of Immunologic Competence of the Host on Metastases Induced by the 3LL Lewis Tumour in Mice. *J. natn. Cancer Inst.*, **52**, 395.

CASTRO, J. E. (1974) Surgical Procedures in Small Laboratory Animals. *J. Immunol. Meth.*, **4**, 213.

CHRISTIE, G. H. & BOMFORD, R. (1975) Mechanisms of Macrophage Activation by *Corynebacterium parvum: In vitro* Experiments. *Cell. Immunol.*, **17**, 141.

CLAMAN, H. N. (1972) Corticosteroids and Lymphoid Cells. *New Engl. J. Med.*, **287**, 388.

CONNING, D. M. & HEPPLESTON, A. G. (1966) Reticuloendothelial Activity and Local Particle Disposal. A Comparison of the Influence of Modifying Agents. *Br. J. exp. Path.*, **47**, 388.

GHAFFAR, A., CULLEN, R. T. & WOODRUFF, M. F. A. (1975) Further Analysis of the Antitumour Effect *In vitro* of Peritoneal Exudate Cells from Mice Treated with *Corynebacterium parvum*. *Cancer, N.Y.*, **31**, 15.

HALPERN, B. N., BIOZZI, G., STIFFEL, C. & MOUTON, D. (1966) Inhibition of Tumour Growth by Administration of Heat Killed *Corynebacterium parvum*. *Nature, Lond.*, **212**, 853.

HALPERN, B. N., PREVOT, A.-R., BIOZZI, G., STIFFEL, C., MOUTON, D., MORARD, J. C., BOUTHILLIER, Y. & DECREUSEFOND, C. (1963) Stimulation de l'Activité Phagocytaire du Système Réticuloendothelial Provoquée par *Corynebacterium parvum*. *J. Reticuloendoth. Soc.*, **1**, 77.

HIBBS, J. B. (1975) Activated Macrophages as Cytotoxic Effector Cells: I. Inhibition of Specific and Non-specific Tumour Resistance by Trypan Blue. *Transplantation*, **19**, 77.

HOWARD, J. G., CHRISTIE, G. H. & SCOTT, M. T. (1973) Biological Effects of *Corynebacterium parvum*: IV. Adjuvant and Inhibitory Activities of B Lymphocytes. *Cell. Immunol.*, **7**, 290.

JAMES, S. E. & SALSBURY, A. J. (1974) Effect of (±) −1, 2-Bis (3,5-dioxopiperazin-1-yl) propane on Tumour Blood Vessels and its Relationship to the Antimetastatic Effect in the Lewis Lung Carcinoma. *Cancer Res.*, **34**, 839.

KAPPLER, J. W., HUNTER, P. C., JACOBS, D. & LORD, E. (1974) Functional Heterogenicity among the Thymus-derived Lymphocytes of the Mouse: 1. Analysis by Adult Thymectomy. *J. Immunol.*, **113**, 27.

KESSEL, R. W. I., MONACO, L. & MARCHISIO, M. A. (1963) The Specificity of the Cytotoxic Action of Silica. A Study *In vitro*. *Br. J. exp. Path.*, **44**, 351.

LANCE, E. M., MEDAWAR, P. B. & TAUB, R. N. (1973) Antilymphocyte Serum. *Advanc. Immunol.*, **17**, 1.

LEVY, M. H. & WHEELOCK, E. F. (1975) Effects of Intravenous Silica on the Immune and Non-immune Functions of the Murine Host. *J. Immunol.*, **115**, 41.

MCBRIDE, W. H., TUACH, S. & MARMION, B. P. (1975) The Effect of Gold Salts on Tumour Immunity and its Stimulation by *Corynebacterium parvum*. *Br. J. Cancer*, **32**, 558.

MILAS, L., HUNTER, N., MASON, K. & WITHERS, H. R. (1974) Immunological Resistance to Pulmonary Metastases in C3Hf/Bu Mice Bearing Syngeneic Fibrosarcoma of Different Sizes. *Cancer Res.*, **34**, 61.

PEARSALL, N. N. & WEISER, R. S. (1968) The Macrophage in Allograft Immunity: I. Effects of Silica as a Specific Macrophage Toxin. *J. Reticuloendoth. Soc.*, **5**, 107.

PROCTOR, J., RUDENSTAM, C. M. & ALEXANDER, P. (1973) Increased Incidence of Lung Metastases Following Treatment of Rats bearing Hepatomas with Irradiated Tumour Cells and the Beneficial Effect of *Corynebacterium parvum* in this System. *Biomed. Express*, **19**, 248.

SADLER, T. E. & CASTRO, J. E. (1976) Abrogation of the Antimetastatic Activity of *Corynebacterium parvum* by Antilymphocyte Serum. *Br. J. Cancer.*, **34**, 291.

SCOTT, M. T. (1975) *In vivo* Cortisone Sensitivity of Non-specific Anti-tumour Activity of *Corynebacterium parvum*-activated Mouse Peritoneal Macrophages. *J. natn. Cancer. Inst.*, **54**, 789.

SIMPSON-HERREN, L., SANFORD, A. H. & HOLMQUIST, J. P. (1974) Cell Population Kinetics of Transplanted and Metastatic Lewis Lung Carcinoma. *Cell Tiss. Kinet.*, **7**, 349.

SMITH, S. E. & SCOTT, M. T. (1972) Biological Effects of *Corynebacterium parvum*: III. Amplification of Resistance and Impairment of Active Immunity to Murine Tumours. *Br. J. Cancer*, **26**, 361.

SUGIURA, K. & STOCK, C. C. (1955) Studies in a Tumour Spectrum: II. The Effect of Phosphoramides on the Growth of a Variety of Mouse and Rat Tumours. *Cancer Res.*, **15**, 38.

THOMPSON, J. & VAN FURTH, R. (1970) The Effect of Glucocorticosteroids on the Kinetics of Mononuclear Phagocytes. *J. exp. Med.*, **131**, 429.

WARR, G. W. & SLJIVIC, V. S. (1974) Origin and Division of Liver Macrophages during Stimulation of the Mononuclear Phagocyte System. *Cell Tiss. Kinet.*, **7**, 559.

WEXLER, H. (1966) Accurate Identification of Experimental Pulmonary Metastases. *J. natn. Cancer Inst.*, **36**, 641.

WIENER, J., COTTRELL, T. S., MARGARETTEN, W. & SPIRO, D. (1967) An Electron Microscopic Study of Steroid Induced Reticuloendothelial Blockade. *Am. J. Path.*, **50**, 187.

WOLMARK, N. & FISHER, B. (1974) The Effect of a Single and Repeated Administration of *Corynebacterium parvum* on Bone Marrow Macrophage Production in Syngeneic Tumour-bearing Mice. *Cancer Res.*, **34**, 2869.

WOODRUFF, M. F. A. & BOAK, J. L. (1966) Inhibitory Effect of Injection of *Corynebacterium parvum* on the Growth of Tumour Transplants in Isogeneic Hosts. *Br. J. Cancer*, **20**, 345.